# SAFA initiates innate immunity against cytoplasmic RNA virus SFTSV infection

**DOI:** 10.1371/journal.ppat.1010070

**Published:** 2021-11-17

**Authors:** Bin-yan Liu, Xue-jie Yu, Chuan-min Zhou

**Affiliations:** State Key Laboratory of Virology, School of Public Health, Wuhan University, Wuhan, P.R. China; Thomas Jefferson University - Center City Campus: Thomas Jefferson University, UNITED STATES

## Abstract

Nuclear scaffold attachment factor A (SAFA) is a novel RNA sensor involved in sensing viral RNA in the nucleus and mediating antiviral immunity. Severe fever with thrombocytopenia syndrome virus (SFTSV) is a bunyavirus that causes SFTS with a high fatality rate of up to 30%. It remains elusive whether and how cytoplasmic SFTSV can be sensed by the RNA sensor SAFA. Here, we demonstrated that SAFA was able to detect SFTSV infection and mediate antiviral interferon and inflammatory responses. Transcription and expression levels of SAFA were strikingly upregulated under SFTSV infection. SAFA was retained in the cytoplasm by interaction with SFTSV nucleocapsid protein (NP). Importantly, SFTSV genomic RNA was recognized by cytoplasmic SAFA, which recruited and promoted activation of the STING-TBK1 signaling axis against SFTSV infection. Of note, the nuclear localization signal (NLS) domain of SAFA was important for interaction with SFTSV NP and recognition of SFTSV RNA in the cytoplasm. In conclusion, our study reveals a novel antiviral mechanism in which SAFA functions as a novel cytoplasmic RNA sensor that directly recognizes RNA virus SFTSV and mediates an antiviral response.

## Author summary

Severe fever with thrombocytopenia syndrome virus (SFTSV) is an RNA virus with a high fatality rate of up to 30%, which replicates exclusively in the cytoplasm. To date, many cytoplasmic RNA sensors were known to recognize SFTSV infection and trigger antiviral immune responses. Nuclear scaffold attachment factor A (SAFA) is a novel nuclear RNA sensor which can sense viral RNA in the nucleus and promote activation of antiviral immunity. However, there are no studies to investigate whether SAFA could detect cytoplasmic RNA virus infection. Here, we reported that SAFA was able to detect RNA virus SFTSV invasion. Under SFTSV infection, SAFA was retained in the cytoplasm and recognized SFTSV infection by interaction with SFTSV nucleocapsid protein (NP) and cytoplasmic SFTSV RNA directly. Importantly, SAFA recruited and promoted the activation of the STING-TBK1 signaling pathway-mediated antiviral immunity to suppress SFTSV infection. This study provides a further acquaintance in SAFA-mediated antiviral immune responses, illustrating the novel role of SAFA in sensing cytoplasmic SFTSV and mediates an antiviral response.

## Introduction

Severe fever with thrombocytopenia syndrome virus (SFTSV) is a severe tick-borne bunyavirus first discovered in China in 2009, and subsequently reported in South Korea, Japan, Vietnam, Pakistan, and Thailand [1–7]. With a mortality rate of up to 30%, SFTSV poses an imminent public health threat [2]. In 2019, the World Health Organization (WHO) has declared SFTS on the list of Blueprint Priority Diseases [8]. In addition, SFTSV is frequently transmitted from person to person through contact with patient blood [9]. SFTSV is a negative-sense single-stranded RNA (ssRNA) virus and has a spherical virion with an envelope containing the large (L) segment encoding the RNA-dependent RNA polymerase (RdRp) that mediates transcription and replication of SFTSV viral genome, the medium (M) segment encoding the envelope glycoproteins Gn and Gc that mediate invasion and assembly of SFTSV, and the small (S) segment encoding the nucleocapsid protein (NP) that mediates formation of the SFTSV ribonucleoprotein (RNP) complex, and non-structural protein (NSs) that mediates the immune escape of SFTSV [1,9–13].

As the foremost line of defense against invading microbial pathogens, pattern recognition receptors (PRRs) are of great importance in defending the host against microbial infection by recognizing pathogen-associated molecular patterns (PAMPs) [14]. To date, multiple innate immune escape strategies of SFTSV have been introduced, which are mainly dependent on SFTSV NSs. Previous studies reported that TANK-binding kinase 1 (TBK1), inhibitor of nuclear factor kappa B kinase subunit epsilon (IKKε), retinoic acid-inducible gene I (RIG-I), signal transducer, and activator of transcription 1 and 2 (STAT1 and STAT2), interferon regulatory factor 3 (IRF3), and IRF7 were sequestered by SFTSV NSs into inclusion bodies to suppress antiviral innate immunity [15–22]. As with other bunyaviruses, SFTSV replication occurs exclusively in the cytoplasm [23], which is detected by cytoplasmic RNA sensors such as RIG-I and melanoma differentiation-associated gene 5 (MDA5) [23,24]. Upon activation, RIG-I and MDA5 recruit mitochondrial antiviral signaling (MAVS) protein, which leads to activation of downstream kinases TBK1/IKKε/IRF3 and ultimately induces production of type I IFNs [25].

Nuclear matrix protein-nuclear scaffold attachment factor A (SAFA), also known as heterogeneous ribonucleoprotein U, was recently identified as a novel nuclear RNA sensor [26–28]. Upon recognition of viral double-stranded RNA (dsRNA), SAFA is oligomerized in the nucleus and functions as a super-enhancer that promotes activation of antiviral responses through interaction with the chromatin remodeling complex [26]. Based on current knowledge, SAFA functions only as a nuclear RNA sensor to trigger an antiviral immune response. The interaction between SAFA and cytoplasmic RNA virus is still unclear [26]. In this study, we used SFTSV as a model and investigated whether SAFA could recognize RNA viral infections in the cytoplasm.

## Results

### 1. SAFA is involved in the infection of SFTSV

**Fig 1:**
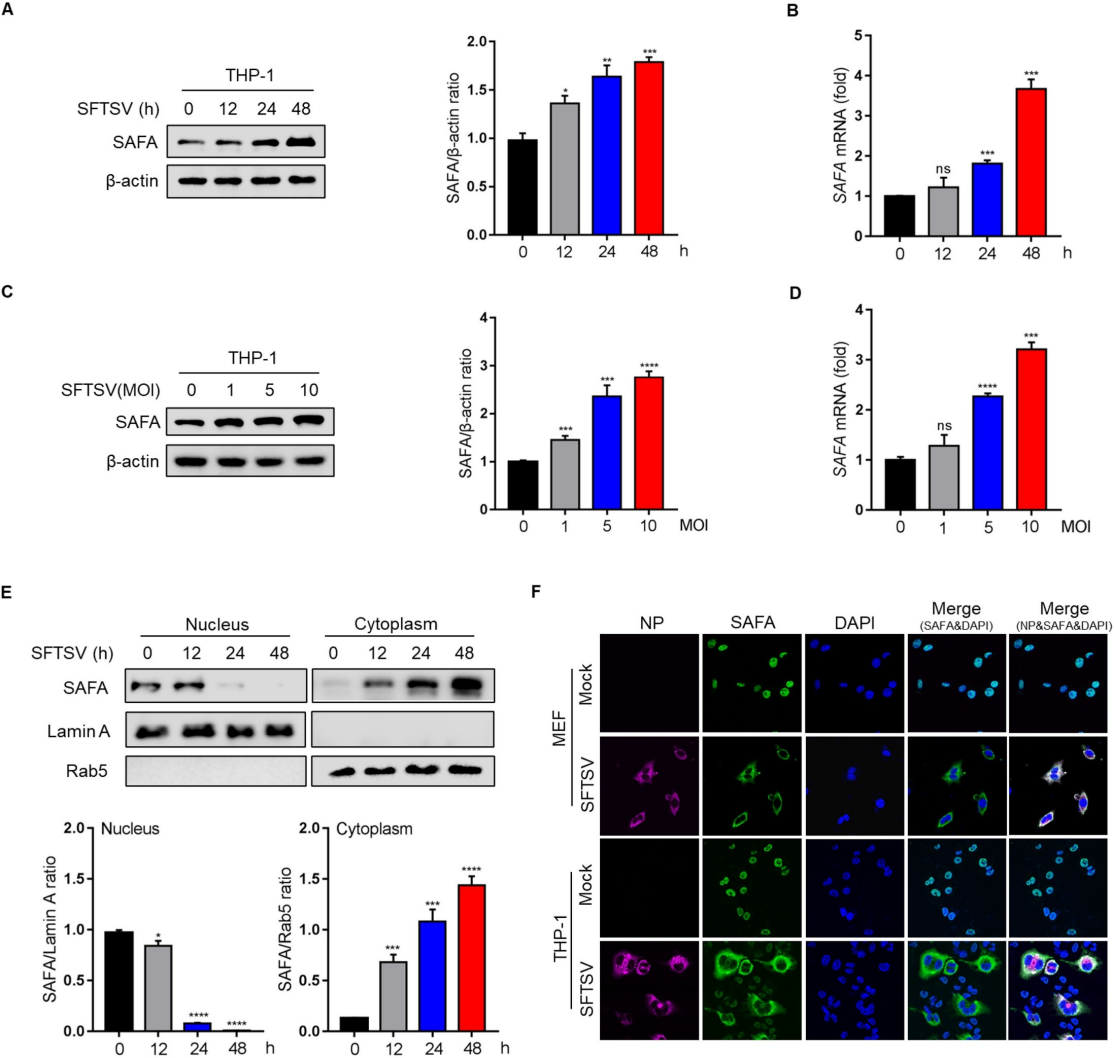
SAFA is involved in the infection of SFTSV. **(A)** THP-1 cells were infected with SFTSV (MOI = 10) for 12, 24, or 48 h. SAFA protein levels were analyzed by Western blot. Western blot data were semi-quantified and normalized against β-actin protein loading control. **(B)** THP-1 cells were infected with SFTSV (MOI = 10) for 12, 24, or 48 h. *SAFA* mRNA was analyzed by RT-PCR. **(C)** THP-1 cells were infected with SFTSV (MOI = 0, 1, 5, 10) for 48 h. SAFA protein levels were analyzed by Western blot. Western blot data were semi-quantified and normalized against β-actin protein loading control. **(D)** THP-1 cells were infected with SFTSV (MOI = 0, 1, 5, 10) for 48 h. *SAFA* mRNA levels were analyzed by RT-PCR. **(E)** MEF cells were infected with SFTSV (MOI = 10) for the indicated time. Nuclear and cytoplasmic proteins were separated. SAFA, Lamin A, and Rab5 protein levels were analyzed by Western blot. Lamin A and Rab5 were nuclear and cytoplasmic index proteins respectively. Nuclear and cytoplasmic Western blot data were semi-quantified and normalized against Lamin A and Rab5 protein loading control respectively. **(F)** MEF cells and THP-1 cells were infected with or without SFTSV (MOI = 10) for 48 h, SFTSV NP (purple), SAFA (green), and DAPI (blue) were analyzed by confocal microscopy. Data were obtained from three independent experiments (n = 3). *P < 0.05, **P <0.001, ***P <0.0001, ****P <0.00001, ns, not significant.

To determine whether SAFA is involved in SFTSV infection, the transcriptional and expression levels of SAFA were determined in a human macrophage cell line THP-1. Interestingly, we found that the protein and mRNA levels of SAFA were significantly increased under SFTSV infection in a time- **(Fig 1A and 1B)** and dose-dependent manner **(Fig 1C and 1D)** without affecting cell viability **(S1A and S1B Fig)**. *SAFA* mRNA levels were also significantly increased in mouse embryonic fibroblast (MEF) cells with increasing multiplication of infection (MOI) of SFTSV **(S1C Fig)**. Moreover, our immunofluorescence assays objectively showed that the immunofluorescence intensity of SAFA was increased in MEF cells under SFTSV infection **(S1D Fig)**. SAFA has been reported as an RNA sensor in the nucleus [26], whereas SFTSV is a cytoplasmic RNA virus. Therefore, we hypothesized that translocation of SAFA might be the critical step for cytoplasmic RNA virus recognition. To determine the distribution of SAFA in MEF cells under SFTSV infection, SAFA proteins were separated from nucleus and cytoplasm to examine the subcellular distribution of SAFA. As with previous reports [26], SAFA was mainly localized in the nucleus in the resting state (**Fig 1E)**, whereas SAFA accumulated in the cytoplasm under SFTSV infection, and almost disappeared in the nucleus 48 h after exposure to SFTSV (**Fig 1E)**. To further confirm the translocation of SAFA, the subcellular localization of SAFA was determined by confocal microscopy 48 h after SFTSV infection. Consistent with immunoblotting data, SAFA accumulated in the cytoplasm but vanished in the nucleus in both MEF and THP-1 cells under SFTSV infection **(Fig 1F)**. These data suggest that nucleocytoplasmic translocation of SAFA may be essential for recognition of SFTSV infection.

### 2. SAFA mediates innate immune responses during SFTSV infection

**Fig 2:**
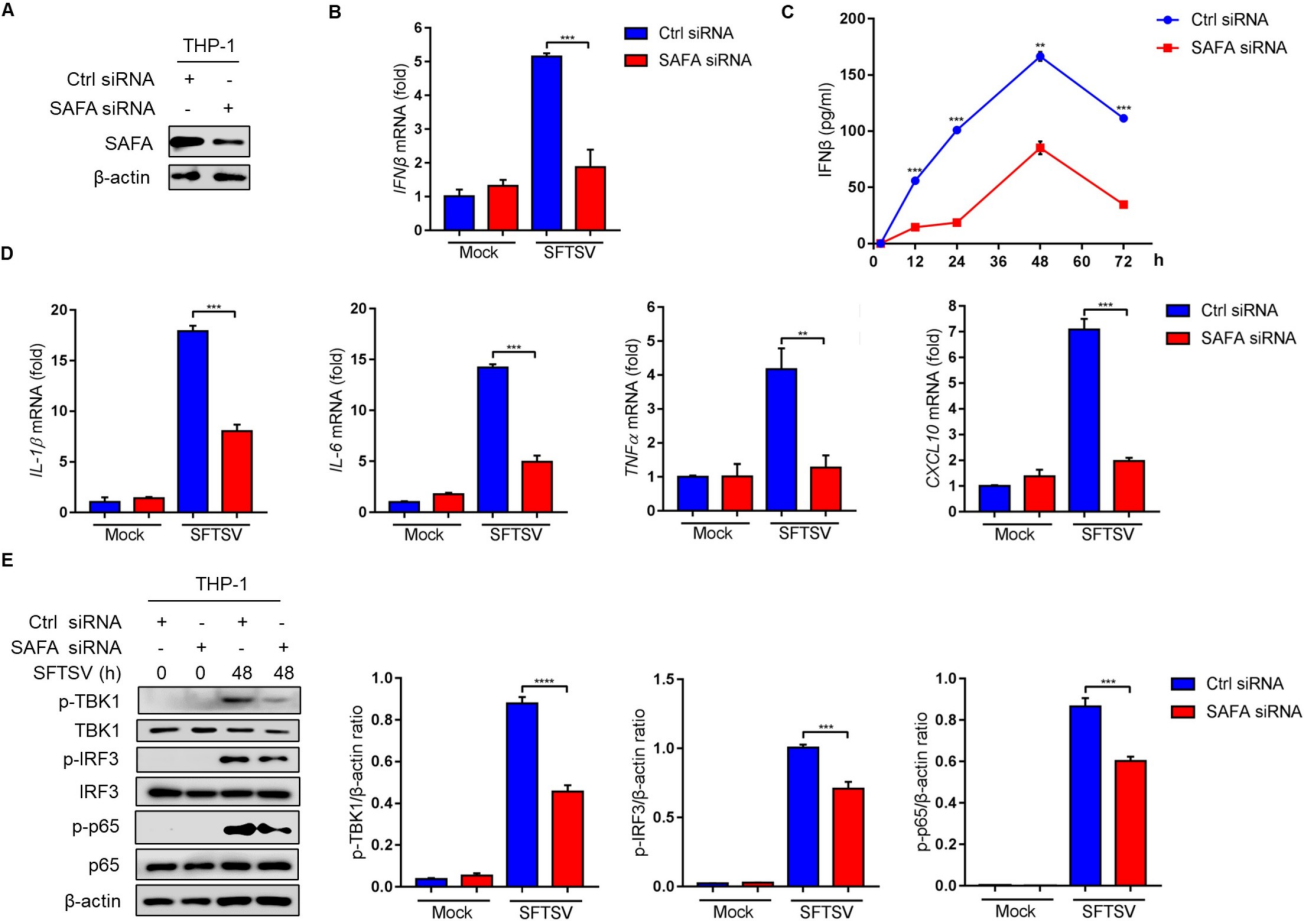
Knockdown of SAFA reduces immune responses induced by SFTSV infection. **(A)** Knockdown of SAFA in THP-1 cells, identified by Western blot. **(B-C)** THP-1 cells were transfected with SAFA siRNA (10 μM, 24 h) or control siRNA (10 μM, 24 h), and then infected with SFTSV (MOI = 10) for 48 h. *IFNβ* mRNA levels were analyzed by RT-PCR **(B)**, IFNβ cytokine levels were analyzed by ELISA**(C)**. **(D)** THP-1 cells were transfected with SAFA siRNA (10 μM, 24 h) or control siRNA (10 μM, 24 h), and then infected with SFTSV (MOI = 10) for 48 h. *IL-1β*, *IL-6*, *TNFα*, and *CXCL10* mRNA levels were analyzed by RT-PCR. **(E)** THP-1 cells were transfected with SAFA siRNA (10 μM, 24 h) or control siRNA (10 μM, 24 h), and then infected with SFTSV (MOI = 10) for 48 h. p-TBK-1, TBK-1, p-IRF3, IRF3, p-p65, and p65 protein levels were analyzed by Western blot. Western blot data were semi-quantified and normalized against β-actin protein loading control. Data were obtained from three independent experiments (n = 3). **P <0.001, ***P <0.0001.

**Fig 3:**
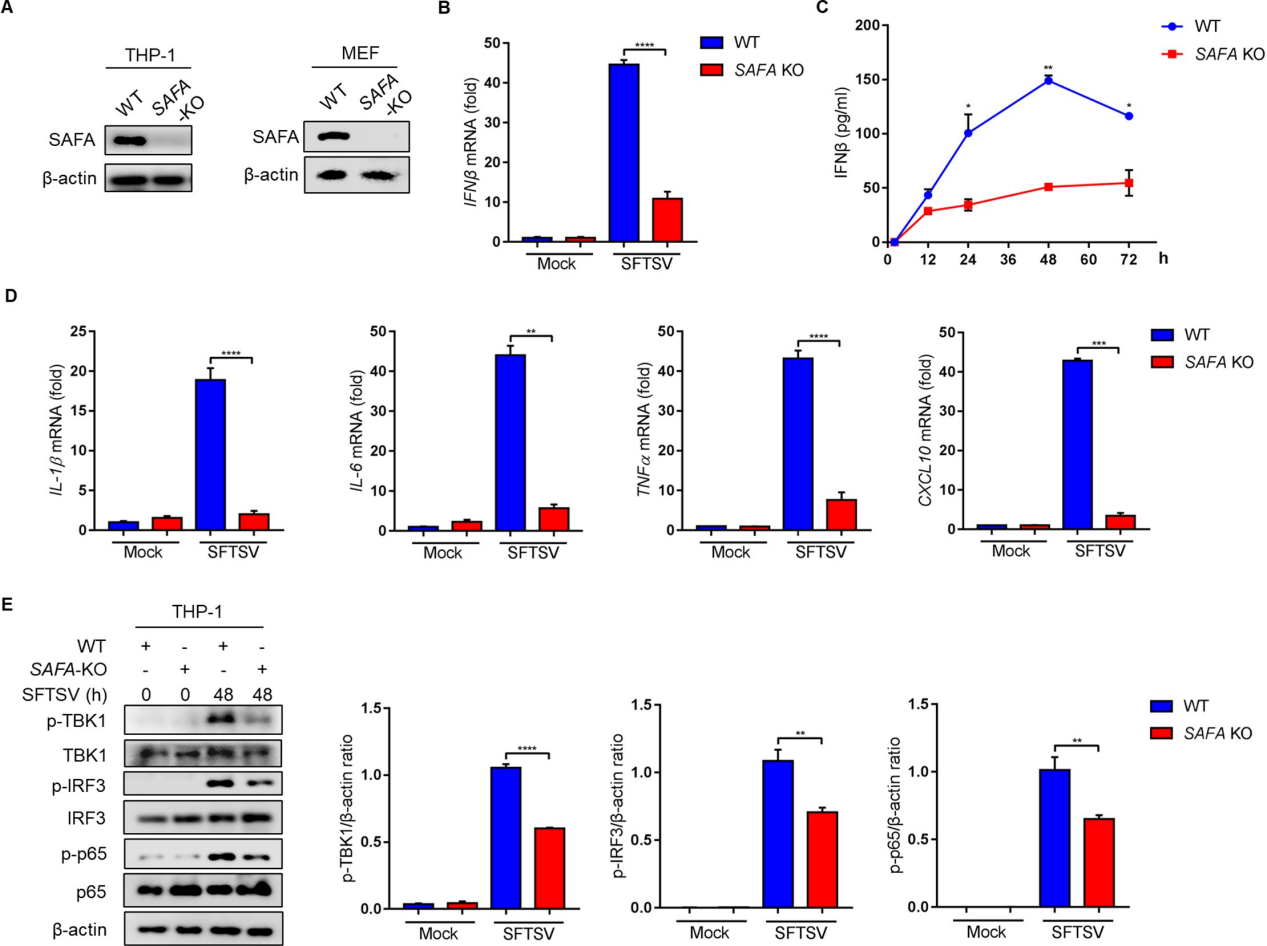
Knockout of SAFA reduces immune responses induced by SFTSV infection. **(A)** Knockout of SAFA in THP-1 cells and MEF cells, identified by Western blot. **(B-C)** WT and *SAFA*^-/-^ MEF cells were infected with SFTSV (MOI = 10) for 48 h, *IFNβ* mRNA level was detected by RT-PCR **(B)**, IFNβ cytokine levels were analyzed by ELISA **(C)**. **(D)** WT and *SAFA*^-/-^ THP-1 cells were infected with SFTSV (MOI = 10) for 48 h, *IL-1β*, *IL-6*, *TNFα*, and *CXCL10* mRNA levels were analyzed by RT-PCR. **(E)** WT and *SAFA*^-/-^ THP-1 cells were infected with SFTSV (MOI = 10) for 48 h. The p-TBK-1, TBK-1, p-IRF3, IRF3, p-p65, and p65 protein levels were analyzed by Western blot. Western blot data were semi-quantified and normalized against β-actin protein loading control. Data were obtained from three independent experiments (n = 3). **P <0.001, ***P <0.0001, ****P <0.00001.

In order to investigate the role of SAFA in innate immunity during SFTSV infection, SAFA expression was knocked down in THP-1 cells using siRNA **(Fig 2A)**. We observed that transfection of SAFA siRNA reduced *IFNβ* transcription **(Fig 2B)** and IFNβ production **(Fig 2C)** after SFTSV infection for 48 h. Correspondingly, transcription of the inflammatory cytokines *IL-1β*, *IL-6*, *TNFα*, and the IFN-stimulated gene *CXCL10* were also reduced in SAFA siRNA-transfected THP-1 cells **(Fig 2D)**. These results indicate that SAFA plays an important role in the induction of type I IFN and inflammatory responses under SFTSV infection. Moreover, the protein levels of phosphorylated TBK1 (p-TBK1), p-IRF3, and p-p65 were analyzed to determine whether SAFA was indeed involved in the activation of type I IFN and inflammatory responses under SFTSV infection. We observed that the protein levels of p-TBK1, p-IRF3, and p-p65 were significantly decreased in SAFA siRNA-transfected THP-1 cells **(Fig 2E and S2A Fig)**. To further characterize the role of SAFA in the response to SFTSV infection, we utilized the CRISPR-Cas9 technique to construct SAFA-KO (*SAFA*^-/-^) THP-1 and MEF cells **(Fig 3A)**. Consistent with siRNA results, *IFNβ* transcription and IFNβ production were significantly decreased in *SAFA*^-/-^ MEF cells **(Fig 3B and 3C)**. In addition, the mRNA levels of *IL-6*, *IL-1β*, *TNFα*, and *CXCL10* induced by SFTSV were decreased in *SAFA*^-/-^ THP-1 cells **(Fig 3D)**, and the protein levels of p-TBK1, p-IRF3, and p-p65 were impaired in *SAFA*^-/-^ THP-1 cells **(Fig 3E)**. These results suggest that SAFA may activate TBK1-mediated type I IFN and inflammatory responses under SFTSV infection.

### 3. SAFA deficiency promotes SFTSV propagation

**Fig 4:**
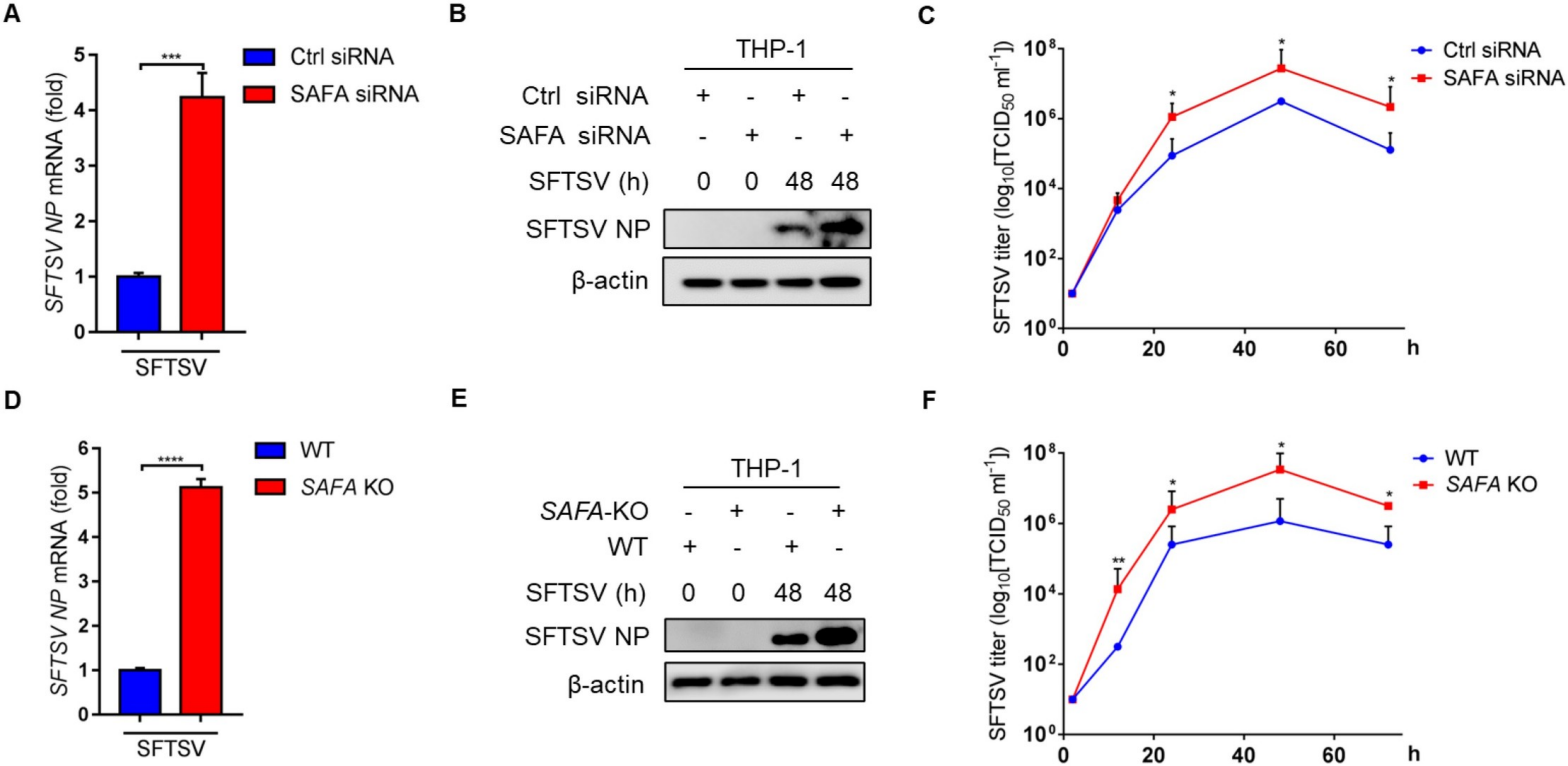
SAFA deficiency promotes SFTSV replication. **(A-B)** THP-1 cells were transfected with SAFA siRNA (10 μM, 24 h) and control siRNA (10 μM, 24 h), and infected with SFTSV (MOI = 10) for 48 h, mRNA **(A)** and protein **(B)** levels of SFTSV NP were analyzed by RT-PCR and Western blot. **(C)** THP-1 cells were transfected with SAFA siRNA (10 μM, 24 h) and control siRNA (10 μM, 24 h), and infected with SFTSV (MOI = 10) for indicated time. SFTSV titers were measured by TCID_50_ assays. **(D-E)** WT and *SAFA*^-/-^ THP-1 cells were infected with SFTSV (MOI = 10) for 48 h, mRNA **(D)** and protein **(E)** levels of SFTSV NP were examined by RT-PCR and Western blot. **(F)** WT and *SAFA*^-/-^ MEF cells were infected with SFTSV for the indicated time. SFTSV titers were measured by TCID_50_ assays. Data were obtained from three independent experiments (n = 3). *P <0.05, **P <0.001.

To determine the effects of SAFA on SFTSV replication, mRNA and protein levels of SFTSV NP were determined in SAFA-deficient THP-1 cells. Interestingly, we observed that both mRNA and protein levels of SFTSV NP were increased in SAFA siRNA-transfected THP-1 cells **(Fig 4A and 4B)** and *SAFA*^-/-^ THP-1 cells **(Fig 4D and 4E)**. Furthermore, median tissue culture infective dose (TCID_50_) was used to detect the exocellular viral titers. We observed that the SFTSV titers were significantly increased in SAFA siRNA transfected THP-1 cells **(Fig 4C)** and *SAFA*^-/-^ MEF cells **(Fig 4F)**. These results suggest that SAFA is a critical factor of innate immunity against SFTSV propagation.

### 4. SFTSV infection drives translocation of SAFA to activate STING-TBK1-mediated antiviral response

**Fig 5:**
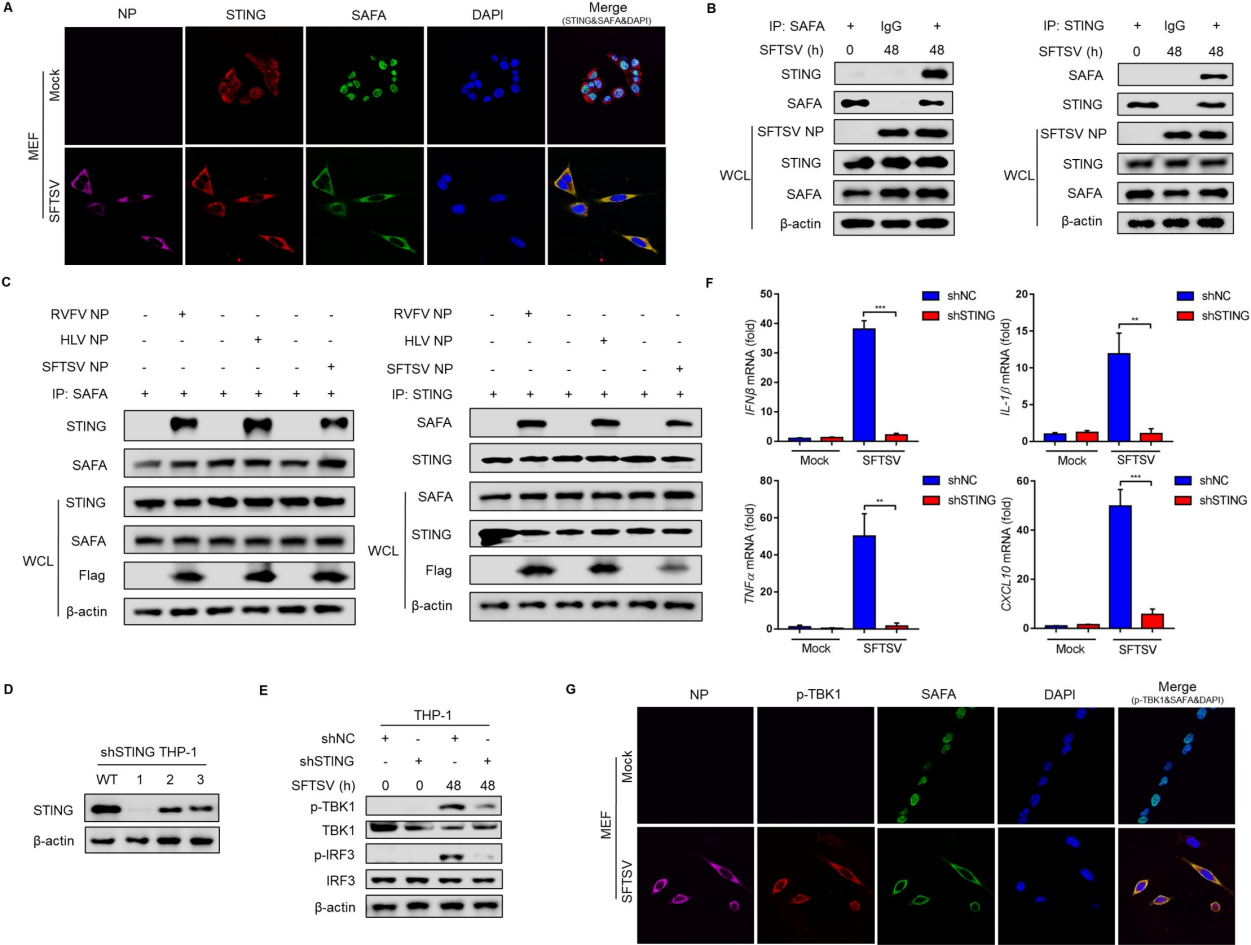
SFTSV infection drives translocation of SAFA to activate the STING-TBK1 mediated antiviral response. **(A)** MEF cells were infected with or without SFTSV (MOI = 10) for 48 h, SFTSV NP (purple), SAFA (green), STING (red), and DAPI (blue) were analyzed by confocal microscopy. **(B)** MEF cells were infected with or without SFTSV (MOI = 10) for 48 h. The interaction between SAFA and STING in MEF cells was analyzed by CO-IP. **(C)** MEF cells were transfected with Flag-tagged RVFV NP, HLV NP or SFTSV NP for 24 h. The interaction between SAFA and STING was analyzed by CO-IP. **(D)** Knockdown of STING in THP-1 cells, analyzed by Western blot. **(E-F)** The shSTING and shNC THP-1 cells were infected with or without SFTSV (MOI = 10) for 48 h. The p-TBK-1, TBK-1, p-IRF3, and IRF3 protein levels were analyzed by Western blot **(E)**, *IFNβ*, *IL-1β*, *TNFα*, and *CXCL10* mRNA levels were analyzed by RT-PCR **(F)**. **(G)** MEF cells were infected with or without SFTSV (MOI = 10) for 48 h. SFTSV NP (purple), SAFA (green), p-TBK1 (red), and DAPI (blue) were analyzed by confocal microscopy.

Many studies suggest that the adaptor protein STING plays a key role in the induction of type I IFN signaling pathway [29,30]. Therefore, we investigated the interaction of SAFA and STING under SFTSV infection. Confocal microscopy revealed that SAFA showed colocalization with STING under SFTSV infection **(Fig 5A)**. Moreover, a co-immunoprecipitation (CO-IP) assay showed that endogenous STING and SAFA could be mutually pulled down in MEF cells under SFTSV infection **(Fig 5B)**. Interestingly, we observed that overexpression of SFTSV NP promoted the interaction of endogenous STING and SAFA in MEF cells **(Fig 5C)**. A similar phenomenon was also observed in MEF cells overexpressed with bunyavirus Rift Valley fever virus (RVFV) NP or Heartland virus (HLV) NP **(Fig 5C)**. To further investigate the role of STING in the immune response under SFTSV infection, we generated stable THP-1 cell lines with specific shRNA targeting STING **(Fig 5D)**. We observed that the p-TBK1 and p-IRF3 were significantly decreased in shSTING THP-1 cells under SFTSV infection **(Fig 5E)**, while the mRNA levels of *IFNβ*, *IL-1β*, *TNFα*, and *CXCL10* were also decreased **(Fig 5F)**. Furthermore, we performed confocal microscopy to analyze the colocalization between SAFA and p-TBK1, the downstream regulator of STING. We found that SAFA could colocalize with p-TBK1 under SFTSV infection **(Fig 5G)**. These data suggest that cytoplasmic SAFA can interact with and activate the STING-TBK1 axis-dependent signaling pathway under SFTSV infection.

### 5. NP is important for the retention of SAFA by interaction with NLS

**Fig 6:**
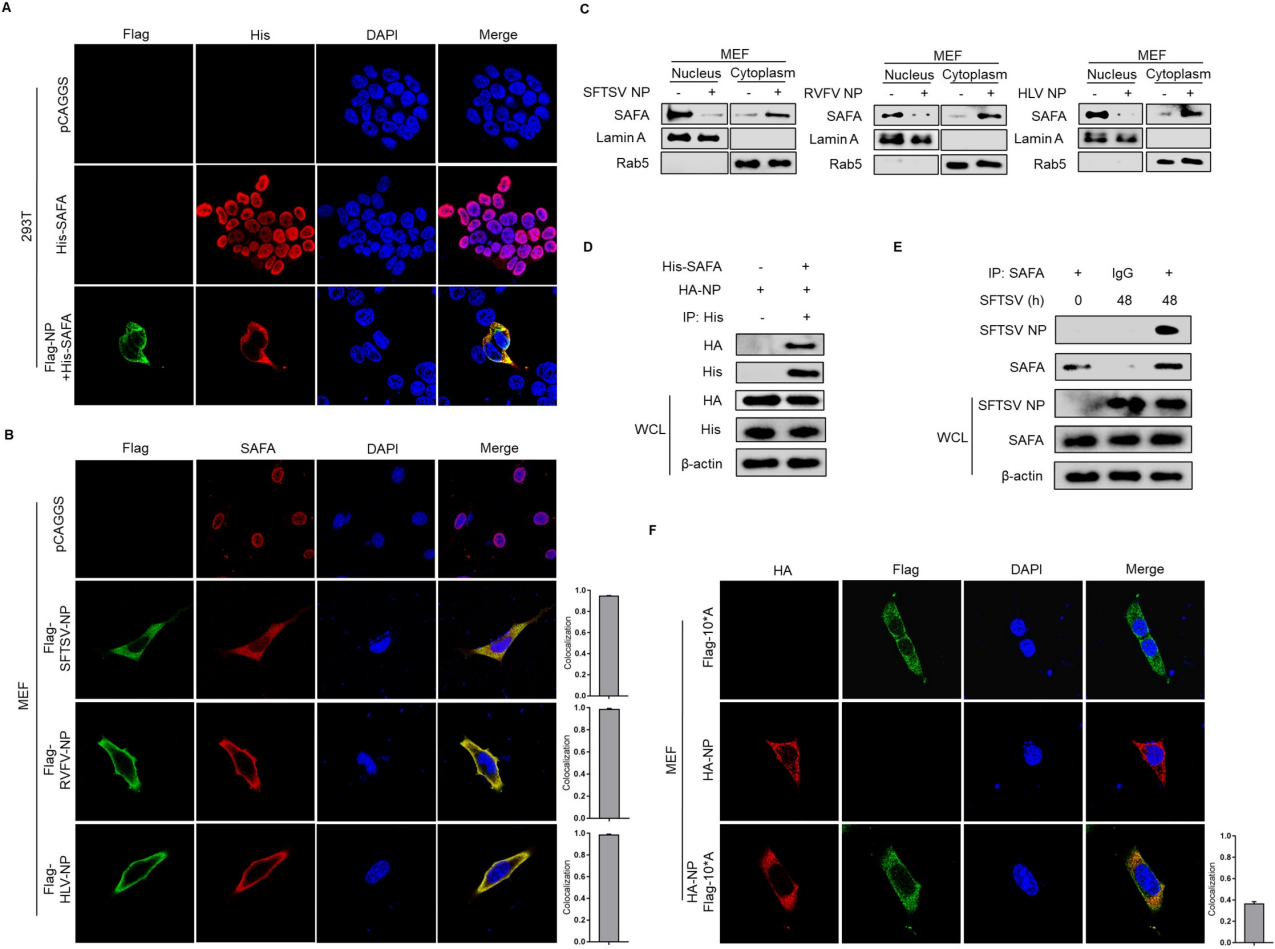
NP is important for retention of SAFA by interaction with NLS. **(A)** HEK293T cells were transfected with the indicated plasmids for 24 h. Flag-tagged SFTSV NP (green), His-tagged SAFA (red), and DAPI (blue) were analyzed by confocal microscopy. **(B)** MEF cells were transfected with the indicated plasmids for 24 h. Flag-tagged SFTSV NP, RVFV NP, HLV NP (green), His-tagged SAFA (red), and DAPI (blue) were analyzed by confocal microscopy. The colocalization of NP and SAFA were quantified. **(C)** MEF cells were transfected with Flag-tagged SFTSV NP, RVFV NP, and HLV NP respectively for 24 h. The nuclear and cytoplasmic protein were separated, SAFA, Lamin A, and Rab5 protein levels were analyzed by Western blot. **(D)** HEK293T cells were transfected with His-tagged SAFA, Flag-tagged SFTSV NP for 24 h, interaction between Flag-tagged NP and His-tagged SAFA was detected by CO-IP. **(E)** MEF cells were infected with or without SFTSV (MOI = 10) for 48h. Interaction between NP and SAFA was analyzed by CO-IP. **(F)** MEF cells were transfected with the indicated plasmids for 24 h, HA-tagged SFTSV NP (red), Flag-tagged 10*A (green), and DAPI were analyzed by confocal microscopy. The colocalization of SAFA and 10*A was quantified.

Considering that SFTSV NP might mediate the interaction between SAFA and the STING-TBK1 axis, we then investigated whether NP can directly interact with SAFA. Interestingly, confocal microscopy revealed that SFTSV NP colocalized with SAFA after SFTSV infection for 48 h in MEF and THP-1 cells **(Fig 1F)**, suggesting that SFTSV NP might be directly involved in the SAFA translocation process. To investigate the underlying interaction mechanism, SAFA and SFTSV NP were overexpressed exogenously in HEK293T cells. Interestingly, we observed that SAFA accumulated in the cytoplasm when co-expressed with SFTSV NP **(Fig 6A)**. Additionally, overexpression of SFTSV NP, RVFV NP, or HLV NP was able to promote the accumulation of SAFA in the cytoplasm in MEF cells **(Fig 6B and 6C**), whereas overexpression of GFP was not **(S3A Fig)**. Moreover, CO-IP assays showed that NP indeed interacted with exogenous SAFA in HEK293T cells **(Fig 6D)** and endogenous SAFA in MEF cells **(Fig 6E)**, indicating the important role of bunyavirus NP in mediating SAFA translocation. Remarkably, transfection of SFTSV genomic RNA failed to promote translocation of SAFA, and the interaction between SAFA and STING **(S3B and S3C Fig)**.

Considering that SFTSV is a cytoplasmic virus, we hypothesized that SFTSV and SFTSV NP might mediate the retention of SAFA by directly interacting with SAFA. It is known that the nuclear localization signal (NLS) domain (240–249 amino acid residues) is critical for mediating nuclear translocation of SAFA. To further investigate whether the NLS domain of SAFA is important for mediating cytoplasmic interaction with NP, a SAFA NLS domain mutant (E240A-R240A) plasmid, namely 10*A, was constructed. Confocal microscopy results showed that 10*A was dispersed in the cytoplasm and showed less colocalization with SFTSV NP **(Fig 6F)**. These data suggest the importance of the SAFA NLS domain in the recognition of SFTSV infection.

### 6. SAFA is involved in recognition of SFTSV NP and RNA in the cytoplasm

**Fig 7:**
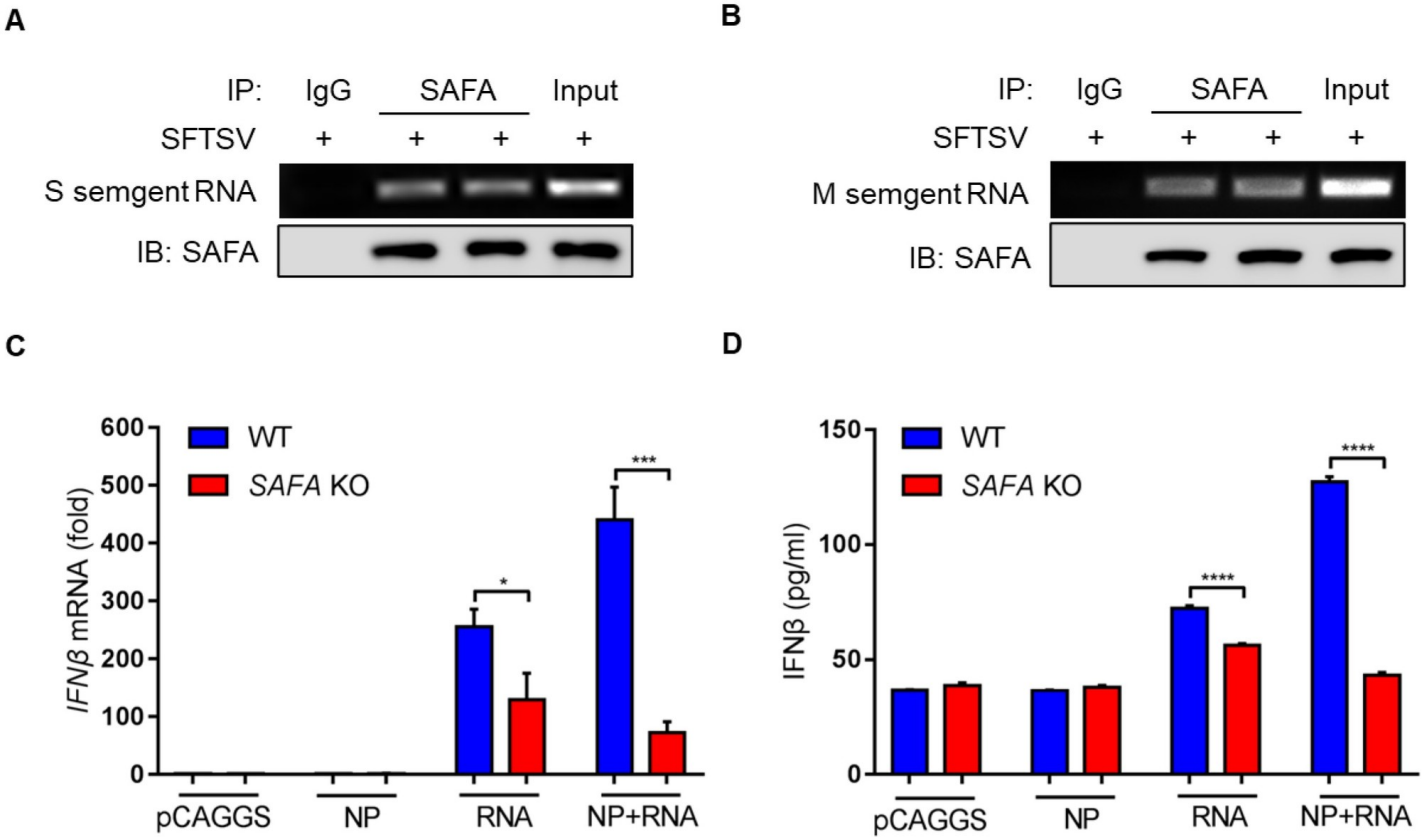
SAFA is involved in detecting SFTSV NP and RNA in the cytoplasm. **(A-B)** MEF cells were infected with or without SFTSV (MOI = 10) for 24 h, interaction between the S **(A)** or M **(B)** segment of SFTSV RNA and SAFA was detected by RIP. **(C-D)** MEF cells were transfected with Flag-tagged SFTSV NP for 24 h, and the purified SFTSV RNA for 6 h or both, *IFNβ* mRNA level was assessed by RT-PCR **(C)**, IFNβ cytokine levels were analyzed by ELISA **(D)**. Data were obtained from three independent experiments (n = 3). *P <0.05, ***P <0.0001, ****P <0.00001.

To further determine whether SAFA can recognize SFTSV RNA, the RNA binding protein immunoprecipitation (RIP) assay was utilized to explore the direct interaction between SAFA and SFTSV RNA. The results showed that both SFTSV S and SFTSV M segment RNA could be pulled down by SAFA under SFTSV infection **(Fig 7A and 7B)**. To assess the role of SFTSV RNA in mediating the activation of signaling cascades, plasmids expressing SFTSV NP and purified SFTSV RNA were transfected into wildtype (WT) and *SAFA*^-/-^ MEF cells. We found that overexpression of SFTSV NP alone barely induced *IFNβ* transcription and IFNβ production, whereas overexpression of SFTSV NP plus SFTSV RNA transformation could promote the *IFNβ* transcription **(Fig 7C)** and IFNβ production **(Fig 7D)**. These data suggest that SAFA may serve as a cytoplasmic RNA sensor in sensing SFTSV NP and RNA and mediating antiviral responses.

## Discussion

In 2019, SAFA was introduced and identified as a novel nuclear RNA sensor that functions in a nuclear-dependent manner. Upon sensing viral RNA in the nucleus, SAFA oligomerizes and interacts with SMARCA5 and TOP1, two key components of the SWI/SNF nucleosome remodeling complex, to promote the activation of enhancers and super-enhancers of antiviral genes. Moreover, SAFA plays an antiviral role in limiting replication of several viruses, including HSV-1, VSV [26], and HIV-1 [31]. However, the role of SAFA in recognizing cytoplasmic RNA viruses remained largely unexplained.

Generally, as a cytoplasmic RNA virus, multiple cytoplasmic RNA PRRs, such as endosomal TLR3 or cytoplasmic RIG-I and MDA5 [32,33], are generally involved in SFTSV recognition. In this study, our data strongly supported the novel role of SAFA as a cytoplasmic RNA sensor in the detection and restriction of cytoplasmic RNA virus SFTSV infection. To date, there has been no report describing the translocation of SAFA after virus infection. Previous studies indicated that SAFA was a purely nuclear protein and did not shuttle between the nucleus and cytoplasm [34,35], which could not induce phosphorylation of IRF3, and stimulated IFN signaling independently of the cytoplasm [26]. Here, we observed that SFTSV promoted translocation of SAFA, whereas SFTSV NP, but not SFTSV RNA, was important for mediating translocation of SAFA. Similar results were also observed with RVFV and HLV NP. Importantly, the NLS domain of SAFA was important for interaction with NP and cytoplasmic translocation, suggesting that SAFA might not be translocated but retained in the cytoplasm under SFTSV infection.

STING has been broadly identified to mediate the activation of innate immune responses [29,36,37]. Here, we illustrated that STING was involved in the recognition of SFTSV infection and mediation of the downstream type I IFN and inflammatory responses. Moreover, SFTSV promoted the interaction of SAFA and the STING-TBK1 axis in the cytoplasm, demonstrating the role of SAFA in mediating the activation of the STING-TBK1 pathway. Importantly, another hnRNP family, hnRNPA2B1, can also translocate to the cytoplasm and initiate type I IFN pathway in a STING-dependent manner [38]. Although SFTSV NP was able to promote the interaction of SAFA and STING in the cytoplasm, SFTSV NP itself was unable to promote the production of type I IFNs. Moreover, we found that SAFA could recognize SFTSV RNA directly. SFTSV NP exhibited synergistic effect that promoted the production of type I IFNs-induced by SFTSV RNA in a SAFA-dependent manner.

Like other bunyaviruses, SFTSV NP is important for the formation of ribonucleoprotein (RNP) complexes and essential for viral replication [39,40]. However, the role of SFTSV NP is largely unclear in immunological studies [41]. Our data extend the role of SFTSV NP and suggest that both of SFTSV NP and SFTSV viral RNA are PAMPs recognized by SAFA. SAFA recognition mechanisms after SFTSV infection might be divided into two main steps: (1) NP directly mediates retention of SAFA by interacting with the NLS domain of SAFA; (2) SAFA recognizes the exogenous SFTSV RNA and then activates STING-TBK1-dependent signaling cascades. Interestingly, we found NSs of SFTSV, as an important virulence factor, can also mediate the translocation of SAFA **(S4A and S4B Fig)**, suggesting that NSs may play a special role in SAFA-mediated immunity. In consideration of the ability of NSs in suppressing antiviral innate immunity [15–22], the relationship between NSs and SAFA could be evaluated in future studies. These results suggest that the interaction between SFTSV and SAFA appears to be more complicated and not solely dependent on SFTSV NP. Other SFTSV components might cooperate with NP in SAFA-mediated antiviral responses or restrict the activation of SAFA for immune escape. The detailed potential function and mechanism need further investigation.

**Fig 8:**
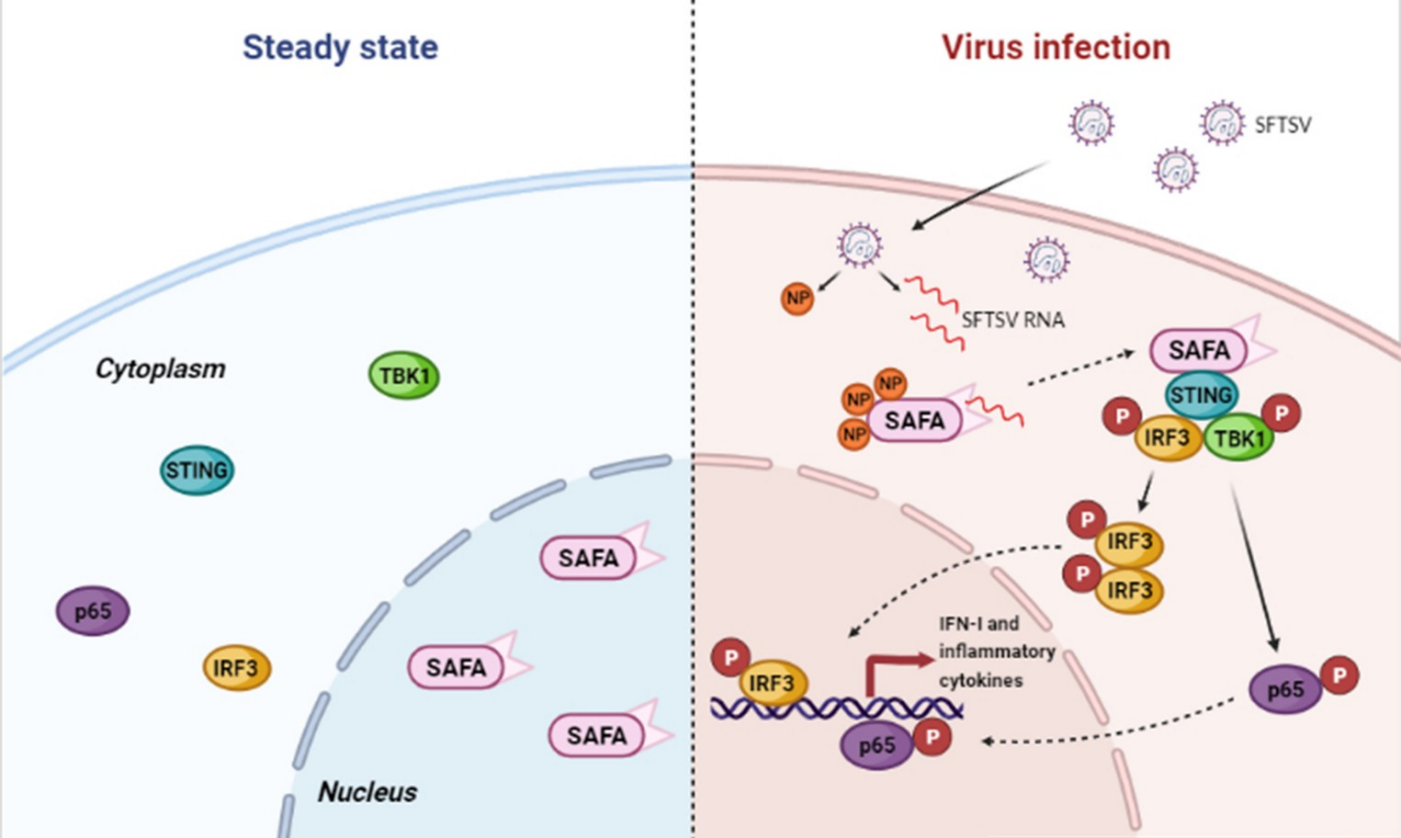
Schematic model illustrating the role of SAFA in sensing cytoplasmic RNA virus SFTSV infection. SAFA remains in the nucleus in the resting state. Under SFTSV infection, SAFA is retained in the cytoplasm by interaction with SFTSV NP. Cytoplasmic SAFA recognizes SFTSV RNA, triggers activation of the STING-TBK1 pathway, and then promotes type I interferon and inflammatory responses against SFTSV infection.

In conclusion, our study extended the novel function of SAFA. SAFA not only detected viral infection in the nucleus, but also directly recognized and restricted the infection of the cytoplasmic RNA virus SFTSV **(Fig 8)**. In addition, our results improve the knowledge of the underlying pathogenicity mechanism of SFTSV, which may provide a theoretical basis for further SFTSV therapies. Considering the diversity of RNA viruses, it is useful to further investigate the spectrum of SAFA in sensing additional RNA viruses and the underlying activation mechanism.

## Materials and methods

### Cells and viruses

Vero, MEF, and HEK293T cells were cultured in DMEM medium (Gibco, Beijing, China) supplemented with 10% fetal bovine serum (FBS; Gibco, Auckland, New Zealand) and 1% streptomycin-penicillin (p/s) at 37°C with 5% CO_2_. Human macrophage cell lines THP-1 were cultured in RPMI 1640 medium (Gibco, Beijing, China) and induced with Phorbol 12-myristate 13-acetate (PMA, 100 ng/ml) for 48 h for cell differentiation. SFTSV (strain JS2011-013-1) was utilized in this study after propagation in Vero cells at a MOI of 0.01. Nuclear and cytoplasmic protein extraction kit (Solarbio, Beijing, China) was utilized in MEF cells for separation of nucleus and cytoplasm after SFTSV infection for 48 h.

### Antibodies

Primary antibodies specific for SAFA (hnRNP U) and β-actin were obtained from Santa Cruz Biotechnology (Dallas, TX). Primary antibodies specific for p-TBK1, TBK-1, and p65 were obtained from Cell Signaling Technology (Beverly, MA). Primary antibodies specific for STING was obtained from Abcam Inc (Cambridge, MA). Primary antibodies specific for SFTSV NP were maintained in our laboratory. Primary antibodies specific to p-IRF3, IRF3, p-p65, Flag-tag, and HA-tag were purchased from Abbkine (Wuhan, China). Primary antibodies specific to His-tag were obtained from Proteintech (Wuhan, China).

### RNA interference and CRISPR/Cas 9 system

The siRNA duplexes were transfected into THP-1 cells for gene silence, human SAFA siRNA was 5’-CGUGGUAGUUACUCAAACATT-3’ for human SAFA and human control siRNA was 5’-TTCTCCGAACGTGTCACGT-3’. The siRNA was transfected using Lipofectamine RNAiMAX (Invitrogen, Carlsbad, CA).

*SAFA*^-/-^ THP-1 cell lines were generated using the CRISPR/Cas9 system, and the gene-specific single-guide RNA (sgRNA) sequence was designed using the online CRISPR Design Tools (https://zlab.bio/guide-design-resources). The human SAFA sgRNA sequence was 5’-CACCGGCTGGAGGAAGAGCATCCTA-3’ and the human control sgRNA sequence was 5’-AAACTAGGATGCTCTTCCTCCAGCC-3’. In brief, after LentiCRISPRv2-SAFA or LentiCRISPRv2-Ctrl, pMD2.G, and psPAX2 were packaged together with polyetherimide (PEI) and co-transfected into HEK293T for 48–60 h. The supernatant was collected for precipitation with PEG8000, and the resuspended lysate was mixed with THP-1 cells containing 5 g/ml polybrene. The selected clonal cells were identified by gene sequencing and Western blot.

### Western blot analysis

For Western blot analysis, protein samples were diluted with RIPA lysis buffer (Beyotime, China), briefly ultrasonicated, separated by 12% SDS-PAGE, and transferred to polyvinylidene difluoride (PVDF) membrane (Millipore, USA). After blocking with 5% non-fat milk in Tris-buffered saline and Tween 20 (TBST), the membrane was incubated with primary antibodies overnight at 4°C and then with HRP-conjugated secondary antibodies. The protein levels were detected by ChemiDoc Touch Imaging System (Bio-Rad) and analyzed by ImageLab software.

### RNA extraction and RT-PCR

TRIzol Reagent (Invitrogen, Carlsbad, CA) was used to isolate RNA. The cDNA was synthesized using High Capacity cDNA Reverse Transcription Kit (Invitrogen, Carlsbad, CA). RT-PCR was performed with specific primers. Relative mRNA concentrations were calculated by the 2^−ΔΔCt^ method, normalizing with β-actin. The primers used were listed in **S1 Table**.

### Immunofluorescence and confocal microscopy

Immunofluorescence assays (IFA) were performed to study the subcellular localization of proteins. THP-1 cells and MEF cells were infected with SFTSV for the indicated time, fixed with 4% paraformaldehyde for 20 min, permeabilized with 0.2% Triton X-100, and blocked with 5% bovine serum albumin for 30 min. The corresponding primary antibodies were incubated overnight at 4°C and fluorescently labeled secondary antibodies were stained for 1 h. The 4’, 6-diamidino-2-phenylindole (DAPI; Beyotime, Shanghai, China) was used to counterstain the nuclei. Cells were observed using Olympus IX73 fluorescent inverted microscope for immunofluorescence and Leica sp8 confocal laser microscope with 63x objective for confocal microscopy. All image analyses were performed using the software Leica Application Suite X.

### Coimmunoprecipitation

To confirm the interaction between SAFA and STING *in vitro*, MEF cells and HEK293T cells were transfected with appropriate plasmids for co-immunoprecipitation. In exogenous verification assays, HEK293T cells were infected with SFTSV for 48 h and co-transfected with plasmids for 24 to 36 h in DMEM containing 2% FBS. In endogenous verification assays, MEF cells were directly infected with SFTSV for 48 h. After incubation, the cells were lysed with the IP cell lysis buffer (Beyotime). The cell lysis was then incubated with specific antibody or IgG as negative control overnight at 4°C. Protein A+G agarose (Beyotime) was then added to the cell lysis and gently rotated at 4°C for 3 h. The mixture was then centrifuged and washed 5 times with PBS. The beads were collected and resuspended with SDS-PAGE loading buffer for Western blotting analysis.

### RNA immunoprecipitation (RIP) assay

RNA immunoprecipitation was performed using the RNA Immunoprecipitation Kit (BersinBio, Guangzhou, China). MEF cells (2 × 10^7^) were infected with SFTSV (MOI = 10) for 24 h, the cell lysate was incubated overnight at 4°C with magnetic protein A/G beads conjugated with SAFA antibody or IgG as a negative control, the immunoprecipitated SAFA and RNA was extracted and analyzed by Western blot and PCR, respectively.

### Statistical analysis

Most experiments were performed at least three times and the statistical analysis was performed using Student’s *t* test or one-way analysis ANOVA with GraphPad Prism Software, where *P *< 0.05 was considered statistically significant.

## Supporting information

S1 Fig The increased SAFA expression and cell viability under SFTSV infection. **(A)** MEF cells were infected with SFTSV (MOI = 10) for 12, 24, or 48 h. Cell viability was analyzed using Cell Counting Kit-8 (CCK8). **(B)** MEF cells were infected with SFTSV (MOI = 0, 1, 5, 10) for 48 h. Cell viability was analyzed using CCK8. **(C)** MEF cells were infected with SFTSV (MOI = 0, 1, 5, 10) for 48 h. *SAFA* mRNA levels were analyzed by RT-PCR. **(D)** Vero cells were infected with SFTSV (MOI = 0, 1, 10) for 24 or 48 h. SAFA protein levels (green) were analyzed by immunofluorescence.
(TIF)

S2 Fig RNAiMAX does not modulate the activation of immune responses under SFTSV infection. **(A)** THP-1 cells were transfected with or without RNAiMAX, after SFTSV infection (MOI = 10) for 48 h, protein levels of p-TBK-1, TBK-1, p-IRF3, IRF3, p-p65, and p65 were examined by Western blot. The protein levels were semi-quantified and levels were normalized by β-actin.
(TIF)

S3 Fig SAFA retains in the nucleus in the absence of SFTSV NP. **(A)** MEF cells were transfected with pmGFP for 24 h. The nuclear and cytoplasmic protein were separated. Expression of SAFA, Lamin A and Rab5 were examined by immunoblot. **(B)** MEF cells were transfected with purified SFTSV RNA for 6 h, the nuclear and cytoplasmic protein was separated, and the expression of SAFA, Lamin A and Rab5 were examined by immunoblot. **(C)** MEF cells were transfected with purified SFTSV RNA for 6 h. The interaction between SAFA and STING in MEF cells was examined by CO-IP.
(TIF)

S4 Fig SFTSV NSs is involved in the retention of SAFA. **(A)** MEF cells were transfected with Flag-tagged SFTSV NSs for 24 h. The nuclear and cytoplasmic protein was separated. SAFA, Lamin A, and Rab5 protein levels were analyzed by Western blot. **(B)** MEF cells were transfected with Flag-tagged SFTSV NSs for 24 h. Flag-tagged SFTSV NSs (green), SAFA (red), and DAPI (blue) were analyzed by confocal microscopy.
(TIF)

S1 Table Primers used for RT-PCR. (DOCX)
