# Supplementary figures and images for: SAFA initiates innate immunity against cytoplasmic RNA virus SFTSV infection
Source: PLoS Pathog. 2021 Nov 17;17(11):e1010070. doi: 10.1371/journal.ppat.1010070 (PMC8598041; doi:10.1371/journal.ppat.1010070)

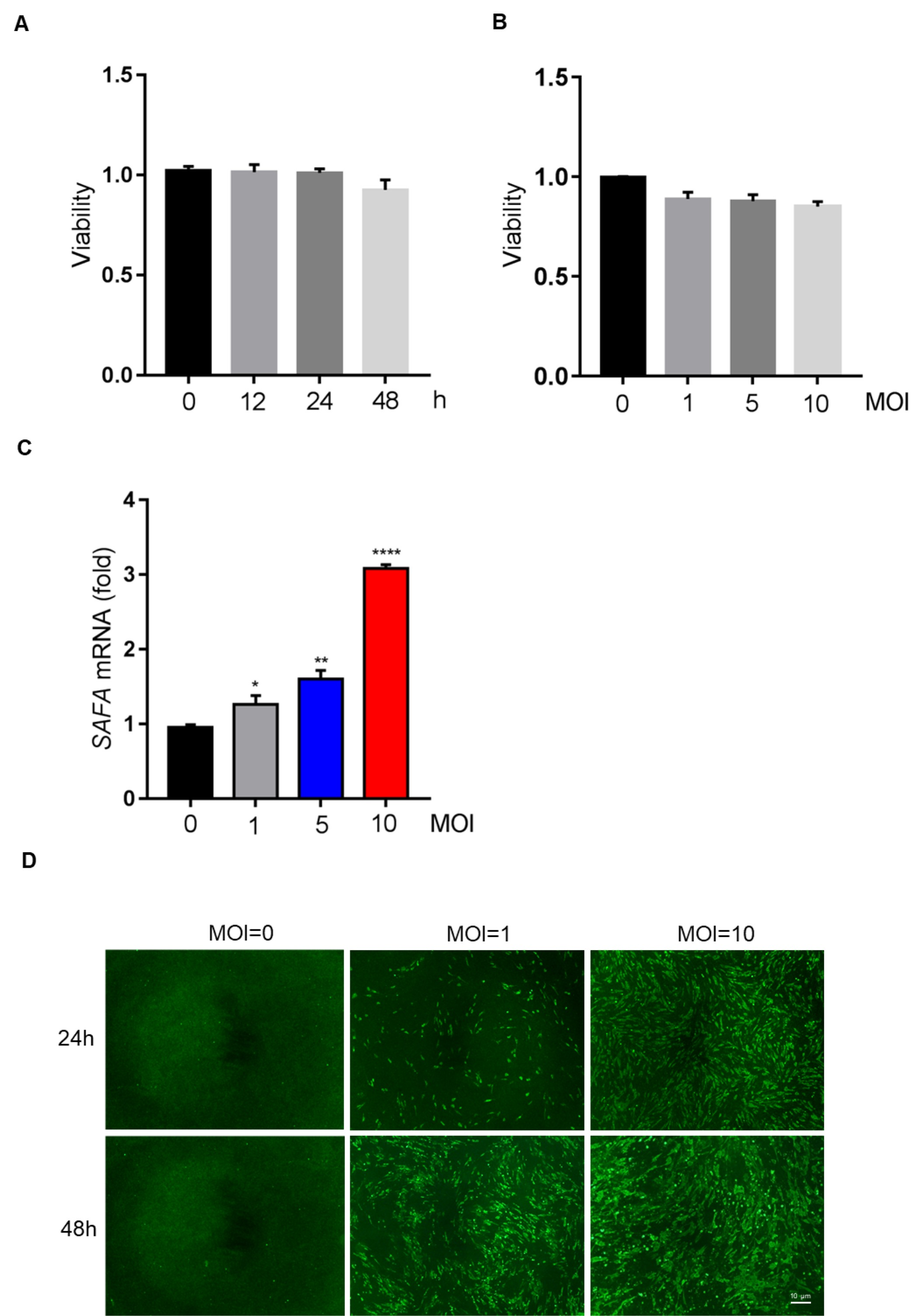

Supplement: S1 Fig — (A) MEF cells were infected with SFTSV (MOI = 10) for 12, 24, or 48 h. Cell viability was analyzed using Cell Counting Kit-8 (CCK8). (B) MEF cells were infected with SFTSV (MOI = 0, 1, 5, 10) for 48 h. Cell viability was analyzed using CCK8. (C) MEF cells were infected with SFTSV (MOI = 0, 1, 5, 10) for 48 h. SAFA mRNA levels were analyzed by RT-PCR. (D) Vero cells were infected with SFTSV (MOI = 0, 1, 10) for 24 or 48 h. SAFA protein levels (green) were analyzed by immunofluorescence. (TIF) [file ppat.1010070.s001.tif]

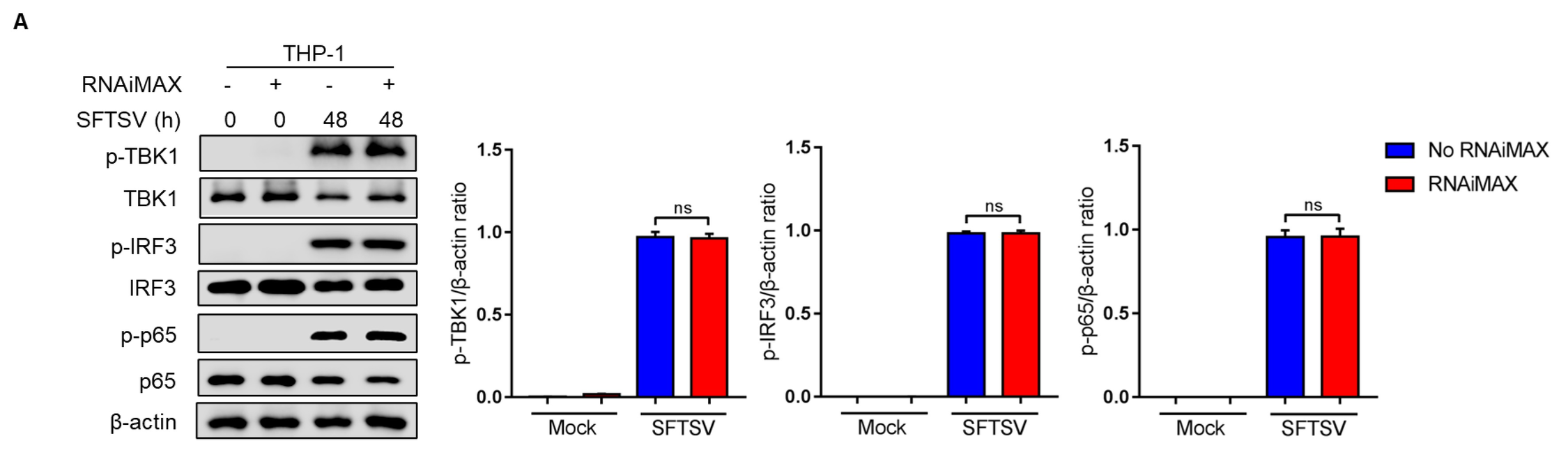

Supplement: S2 Fig — (A) THP-1 cells were transfected with or without RNAiMAX, after SFTSV infection (MOI = 10) for 48 h, protein levels of p-TBK-1, TBK-1, p-IRF3, IRF3, p-p65, and p65 were examined by Western blot. The protein levels were semi-quantified and levels were normalized by β-actin. (TIF) [file ppat.1010070.s002.tif]

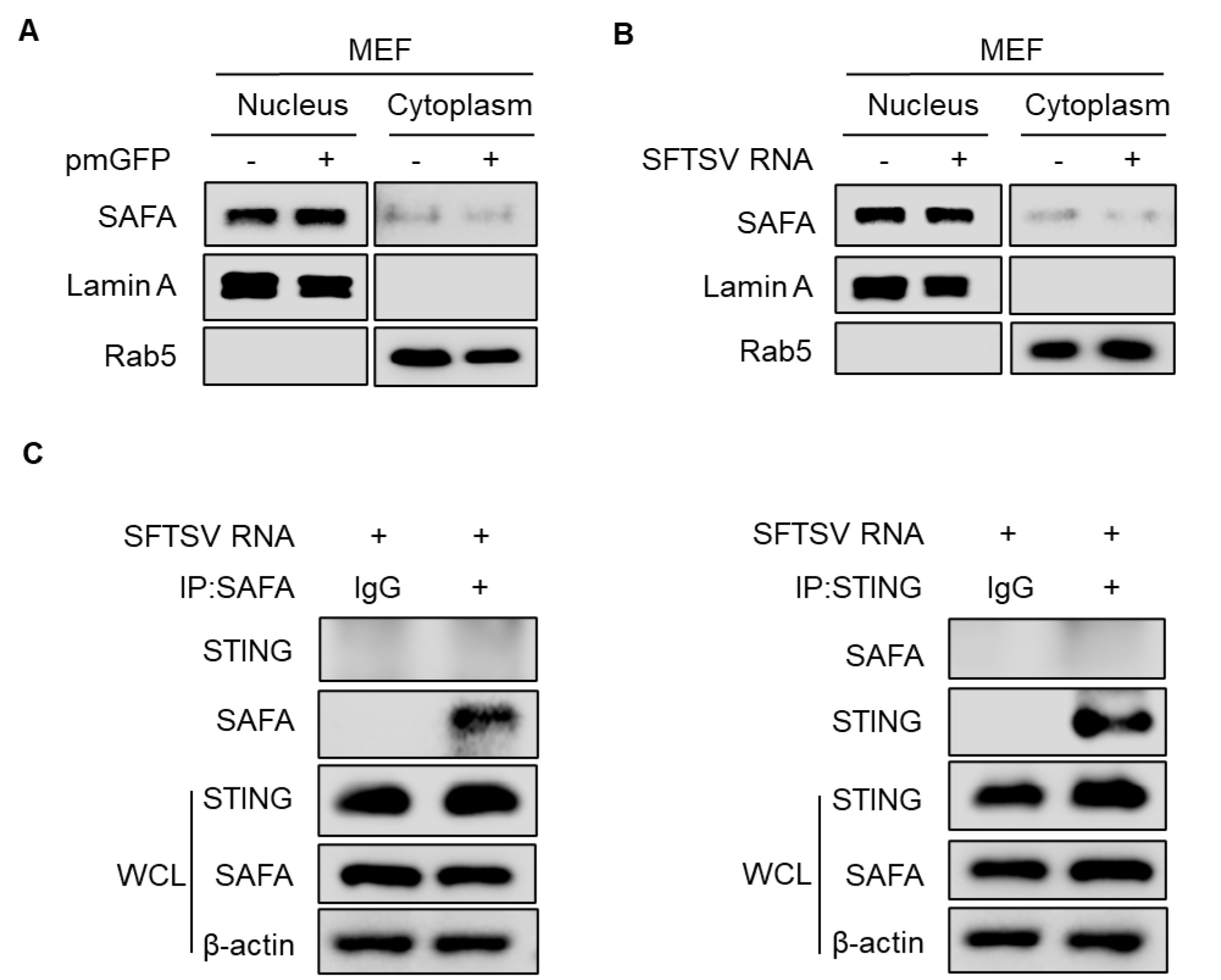

Supplement: S3 Fig — (A) MEF cells were transfected with pmGFP for 24 h. The nuclear and cytoplasmic protein were separated. Expression of SAFA, Lamin A and Rab5 were examined by immunoblot. (B) MEF cells were transfected with purified SFTSV RNA for 6 h, the nuclear and cytoplasmic protein was separated, and the expression of SAFA, Lamin A and Rab5 were examined by immunoblot. (C) MEF cells were transfected with purified SFTSV RNA for 6 h. The interaction between SAFA and STING in MEF cells was examined by CO-IP. (TIF) [file ppat.1010070.s003.tif]

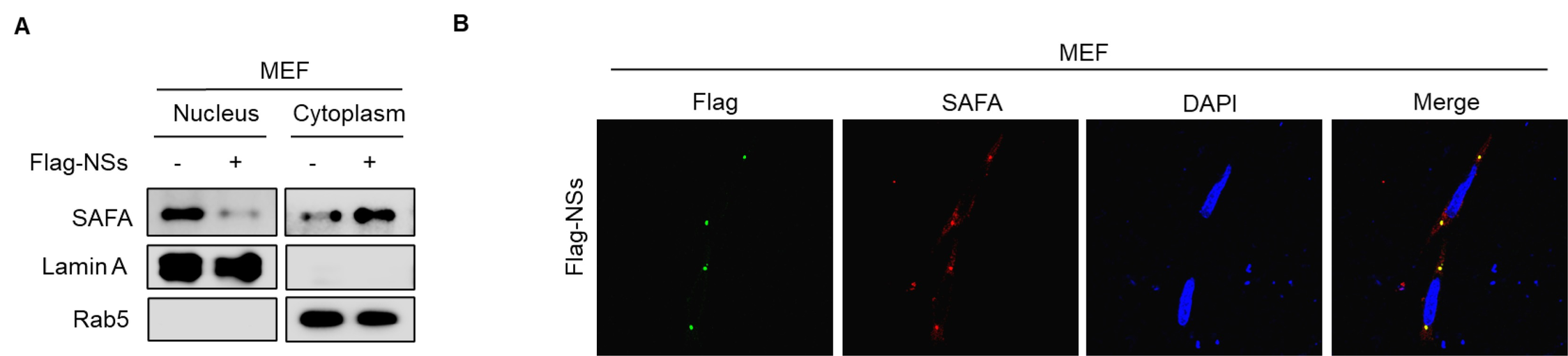

Supplement: S4 Fig — (A) MEF cells were transfected with Flag-tagged SFTSV NSs for 24 h. The nuclear and cytoplasmic protein was separated. SAFA, Lamin A, and Rab5 protein levels were analyzed by Western blot. (B) MEF cells were transfected with Flag-tagged SFTSV NSs for 24 h. Flag-tagged SFTSV NSs (green), SAFA (red), and DAPI (blue) were analyzed by confocal microscopy. (TIF) [file ppat.1010070.s004.tif]
