# Supplementary material for: SAFA initiates innate immunity against cytoplasmic RNA virus SFTSV infection
Source: PLoS Pathog. 2021 Nov 17;17(11):e1010070. doi: 10.1371/journal.ppat.1010070 (PMC8598041; doi:10.1371/journal.ppat.1010070)
Supplement: S1 Table — (DOCX) [file ppat.1010070.s005.docx]

S1 Table. Primers used for RT-PCR.

| Primer name | Forward primer sequence (5’-3’) | Reverse primer sequence (5’-3’) |
| --- | --- | --- |
| Human SAFA | AGGAAGTTCTTGCTGGACGG | GGCCCCTTTGGTCCTCTAAC |
| Human IFNβ | CTTCTCCACTACAGCTCTTTCC | GCCAGGAGGTTCTCAACAATA |
| Human IL-1β | CCAGCTACGAATCTCCGACC | CATGGCCACAACAACTGACG |
| Human IL-6 | TTCGGTCCAGTTGCCTTCTC | TCTTCTCCTGGGGGTACTGG |
| Human TNFα | CCTCTCTCTAATCAGCCCTCTG | GAGGACCTGGGAGTAGATGAG |
| Human β-actin | GACCACCTTCAACTCCATCAT | CCTGCTTGCTAATCCACATCT |
| Murine SAFA | AAGGAGGAGCTCAAGAAGCG | CTCCTGCCTCGTTGTCCAG |
| Murine IFNβ | TCCGAGCAGAGATCTTCAGGAA | TGCAACCACCACTCATTCTGAG |
| Murine IL-1β | ACTGTTTCTAATGCCTTCCC | ATGGTTTCTTGTGACCCTGA |
| Murine IL-6 | TCTGCAAGAGACTTCCATCCAGTTGC | AGCCTCCGACTTGTGAAGTGGT |
| Murine CXCL10 | CCTGCCCACGTGTTGAGAT | GAGTCACAGACCCGTCCCTA |
| Murine TNFα | GGCAGCTGGAATCTCTGAAA | CTGCAGGTGTGTCTGCTGAT |
| Murine β-actin | AGAGGGAAATCGTGCGTGAC | CAATAGTGATGACCTGGCCGT |
| SFTSV NP | TGTCAGAGTGGTCCAGGATT | ACCTGTCTCCTTCAGCTTCT |
| SFTSV M segment | AAGAAGTGGCTGTTCATCATTATTG | GCCTTAAGGACATTGGTGAGTA |
